# A comparison of estimates of glomerular filtration in critically ill patients with augmented renal clearance

**DOI:** 10.1186/cc10262

**Published:** 2011-06-08

**Authors:** João Pedro Baptista, Andrew A Udy, Eduardo Sousa, Jorge Pimentel, Lisa Wang, Jason A Roberts, Jeffrey Lipman

**Affiliations:** 1 Serviço de Medicina Intensiva, Hospitais da Universidade de Coimbra, EPE Praceta Prof. Mota Pinto, Av. Bissaya Barreto 3000-075 Coimbra, Portugal; 2 Department of Intensive Care Medicine, Royal Brisbane and Women's Hospital, Butterfield Street, Herston 4029, Queensland, Australia; 3 Burns, Trauma, and Critical Care Research Center, University of Queensland, Royal Brisbane and Women's Hospital, Butterfield Street, Herston 4029, Queensland, Australia

## Abstract

**Introduction**

Increasingly, derived estimates of glomerular filtration, such as the modification of diet in renal disease (MDRD) equation and Cockcroft-Gault (CG) formula are being employed in the intensive care unit (ICU). To date, these estimates have not been rigorously validated in those with augmented clearances, resulting in potentially inaccurate drug prescription.

**Methods**

Post-hoc analysis of prospectively collected data in two tertiary level ICU's in Australia and Portugal. Patients with normal serum creatinine concentrations manifesting augmented renal clearance (ARC) (measured creatinine clearance (CL_CR_) > 130 ml/min/1.73 m^2^) were identified by chart review. Comparison between measured values and MDRD and CG estimates were then undertaken. Spearman correlation coefficients (r_s_) were calculated to determine goodness of fit, and precision and bias were assessed using Bland-Altman plots.

**Results**

Eighty-six patients were included in analysis. The median [IQR] measured CL_CR _was 162 [145-190] ml/min/1.73 m^2^, as compared to 135 [116-171], 93 [83-110], 124[102-154], and 108 [87-135] ml/min/1.73 m^2 ^estimated by CG, modified CG, 4-variable MDRD and 6-variable MDRD formulae. All of the equations significantly under-estimated the measured value, with CG displaying the smallest bias (39 ml/min/1.73 m^2^). Although a moderate correlation was noted between CL_CR _and CG (r_s _= 0.26, *P *= 0.017) and 4-variable MDRD (r_s _= 0.22, *P *= 0.047), neither had acceptable precision for clinical application in this setting. CG estimates had the highest sensitivity for correctly identifying patients with ARC (62%).

**Conclusions**

Derived estimates of GFR are inaccurate in the setting of ARC, and should be interpreted with caution by the physician. A measured CL_CR _should be performed to accurately guide drug dosing.

## Introduction

Accurate assessment of renal function in the critically ill is essential, not only to detect acute kidney injury, but also for the appropriate prescription of pharmaceuticals and timely application of therapeutic strategies. Although the kidneys have a range of functions in normal homeostasis, the glomerular filtration rate (GFR) remains the most widely accepted index of renal function in both health and disease [1]. Largely, any assessment of GFR in clinical practice focuses on identifying renal impairment, where serum creatinine concentrations are typically employed as a key biomarker for this purpose. In respect to drug prescription, elevated serum creatinine concentrations regularly trigger dose reduction for renally excreted drugs, although the converse-increasing drug dosing in response to low serum values-is infrequently considered in clinical practice.

To further improve the sensitivity of such measures to screen for and monitor chronic kidney disease (CKD), Levey and colleagues have developed a formula to estimate the glomerular filtration rate (eGFR) from serum creatinine concentrations and readily available demographic variables [2]. Although initially developed in a cohort of ambulatory out-patients with CKD, the modification of diet in renal disease (MDRD) equation has been widely adopted in clinical practice, and is now routinely reported by laboratories worldwide. In particular, there has been an increasing trend to use such measures to modify drug dosing, although concerns have been raised about such practice [3]. Perhaps a more familiar estimate of renal function in optimising drug dosing is that defined by the Cockcroft-Gault equation. Initially described in 1976 in a small cohort of male patients [4], this equation has been widely employed as a surrogate of GFR in both clinical and research practice, although its role in the critically ill remains uncertain.

Importantly, these mathematical estimates fail to consider the important effects of the underlying disease process and additional therapies provided, both of which may significantly alter renal function from baseline. Although ideal filtration markers (such as inulin) have been employed in a research setting, they are infrequently available in clinical practice. Similarly, radio-nucleotide measures of GFR are expensive and impractical in the ICU. As such, a measured renal creatinine clearance (CL_CR_) is possibly the easiest and most accurate measure of GFR routinely available to the intensive care clinician.

Given the established concerns regarding the use of estimates of GFR in the critically ill [3], this *post-hoc *investigation was aimed at characterising the accuracy of four commonly used equations in comparison with a measured CL_CR _in a sub-group of patients exhibiting augmented renal clearance (ARC) or 'supra-normal filtration'. The primary end-point was the precision and bias of these estimates compared with CL_CR _measures.

## Materials and methods

### Study population

This study represents a *post-hoc *analysis of prospectively collected data from two multi-disciplinary tertiary level ICUs in Portugal (20 beds) and Australia (30 beds). The only major patient groups not represented include: paediatric, postoperative cardiac surgical patients and solid organ transplant recipients. Patients enrolled in prospective antibacterial pharmacokinetic studies undertaken between 2005 and 2009 at each centre were eligible for inclusion. All patients had to display normal renal function, determined by serum creatinine concentrations less than 1.4 mg/dl (124 μmol/l), without the requirement for renal replacement therapy. Informed consent was obtained from all participants or a surrogate decision maker, and institutional ethics approval was provided at each facility (Australia: Royal Brisbane and Women's Hospital Human Research Ethics Committee, References 2005/038, 2005/072, 2007/188, and Portugal: Innovation and Development Unit, Coimbra University Hospital, Reference 23/IDU/09/A). From this cohort, a sub-group of patients demonstrating ARC (measured CL_CR _>130 ml/min/1.73 m^2^) were identified. Standard definitions for SIRS, sepsis, severe sepsis or septic shock were employed [5]. Diagnostic groups included trauma, sepsis, respiratory failure without sepsis, post-operative patients without sepsis and others.

### Measurement of CL_CR _and calculation of mathematical estimates

An 8-hour renal creatinine clearance was utilised in Australia, while a 24-hour collection was employed in Portugal, representing differing practice at each institution. This technique involves a standard urinary collection (via an indwelling catheter) for the defined time period, following which the creatinine concentration is measured in both urine and blood. The measured CL_CR _is then calculated according to the equations presented in Table 1. Both centres employ automated analysers using a modified Jaffe technique (alkaline picrate). Reported reference ranges for serum creatinine concentrations are 0.6-1.3 mg/dl (53-115 μmol/l) in Portugal, and 0.8-1.2 mg/dl (73-108 μmol/l) in Australia. The mathematical estimates of GFR chosen for comparison included: Cockcroft-Gault (CG), modified CG, 4-variable and 6-variable MDRD formulae (Table 1). As the studies were conducted prior to implementation of an isotope dilution mass spectrometry (IDMS) traceable assay, the original '186' 4-variable MDRD equation was employed (see Table 1).

**Table 1:** Calculations employed

Formulae
24 hour CL_CR _= (U_CR _× U_Vol_/S_CR _× 1440) × 1.73/BSA
8 hour CL_CR _= (U_CR _× U_Vol_/S_CR _× 480) × 1.73/BSA
BSA = 0.007184 × (Ht)^0.725 ^× (Wt)^0.425^
CG CL_CR _= (140-Age) × Wt × 1.73/(S_CR _× 72 × BSA) × 0.85 if female
Modified CG CL_CR _= if S_CR _<1, use 1
4-variable MDRD eGFR = 186 × S_CR _^-1.154 ^× age^-0.203 ^× 1.210 if black × 0.742 if female
6-variable MDRD eGFR = 170 × S_CR _^-0.999 ^× BUN^-0.17 ^× S_Alb _^0.318 ^× Age^-0.176 ^× 1.18 if black × 0.762 if female

BSA, body surface area (m^2^); BUN, blood urea nitrogen (mg/dl); CG CL_CR_, Cockcroft-Gault creatinine clearance (ml/min/1.73 m^2^); CL_CR _, creatinine clearance (ml/min/1.73 m^2^); eGFR, estimated glomerular filtration rate (ml/min/1.73 m^2^); Ht, height (cm); MDRD, modification of diet in renal disease; S_Alb_, serum albumin concentration (g/dl); S_CR_, serum creatinine concentration (mg/dl); U_CR_, urinary creatinine concentration (mg/dl); U_Vol_, urinary volume (ml); Wt, weight (Kg).

### Statistical analysis

Data are presented as the mean (SD) or median [IQR] as appropriate. Correlations were assessed using a scatter graph and Spearman correlation coefficient (r_s_). A Wilcoxon Signed Rank test was used to compare paired data, where as one-way ANOVA, and Kruskal-Wallis were used for sub-group analysis. Precision and bias were assessed using a Bland-Altman plot, with the bias representing the mean difference between each variable, and precision being one SD from the mean. Statistical significance was defined as a p-value < 0.05, and all statistical analysis employed SPSS 13.0^® ^(SPSS, Chicago, IL) and MedCalc 9.3.8 for Windows^® ^(MedCalc, Mariakerke, Belgium).

## Results

Two hundred and nine patients in total were enrolled in studies at each centre. Demographic details of these cohorts are provided in Table 2. Of these, 86 (Australia *n *= 43, Portugal *n *= 43) were identified as manifesting ARC (CL_CR _> 130 ml/min/1.73 m^2^). Demographic and therapy specific data for this sub-group are also presented. All patients manifesting ARC (*n *= 86) demonstrated a systemic inflammatory response syndrome (SIRS) or sepsis on the day of measurement, with a maximum serum creatinine concentration of 1.26 mg/dl (111 μmol/l) being recorded. Of the patients, 58% were admitted after a trauma, 27% with sepsis, 7% with respiratory failure without sepsis, 3.5% were post-surgical without sepsis and 4.7% had another diagnosis (see Table 2).

**Table 2:** Demographic data

Variable	Portugal (*n *= 120)	Australia (*n *= 89)
Male/Female, n (%)	87 (72.5)/33 (27.5)	64 (71.9)/25 (28.1)
Age, years, mean (SD)	55.9 (21.1)	40.0 (18.9)
APACHE II, mean (SD)	17.2 (6.1)	18.2 (7.4)
Diagnosis, n (%)		
Trauma	56 (46.7)	40 (44.9)
Sepsis	38 (31.7)	39 (43.8)
Respiratory failure (without sepsis)	13 (10.8)	2 (2.2)
Post-operative (without sepsis)	7 (5.8)	3 (3.4)
Other	6 (5.0)	5 (5.6)
	**ARC Subgroup (*n *= 86)**	
Male/Female, n (%)	66 (76.7)/20 (23.3)	
Age, years, median (IQR)	35 (25-51.2)	
Weight, kg, median (IQR)	80 (70-90)	
Height, m, median (IQR)	1.7 (1.68-1.76)	
BSA, m^2^, median (IQR)	1.93 (1.81-2.07)	
APACHE II, mean (SD)	14.8 (5.8)	
SIRS (on day of study), n (%)	86 (100)	
Septic (on day of study), n (%)	65 (75.6)	
Mechanical ventilation (on day of study), n (%)	83 (96.5)	
Vasoactive drugs (on day of study), n (%)	24 (27.9)	
Diuretic (on day of study), n (%)	35 (40.7)	
Fluid balance (on day of study), ml, mean (SD)	311 (1640)	
Serum creatinine, mg/dl (μmol/l), median (IQR)	0.7 (0.6-0.9) (62 (53-80))	
Measured CL_CR_, ml/min/1.73 m^2^, median (IQR)	162 (145-190)	

APACHE, acute physiology and chronic health evaluation; ARC, augmented renal clearance; BSA, body surface area; CL_CR_, creatinine clearance; IQR, interquartile range; SD, standard deviation; SIRS, systemic inflammatory response syndrome.

**Figure 1:**
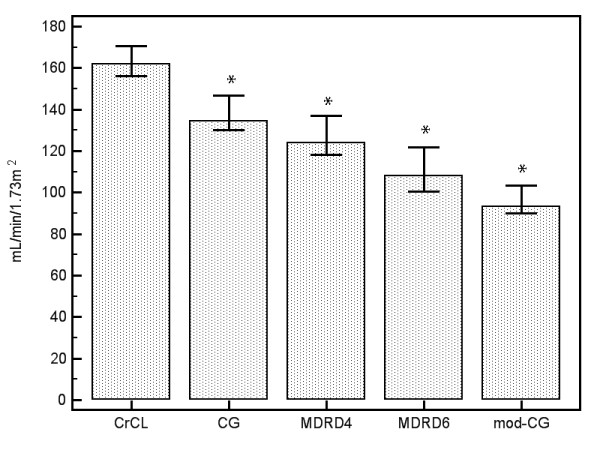
**Comparison of median measured and estimated glomerular filtration rate**. Median values (95% confidence interval) for measured and estimated glomerular filtration rate. All mathematical equations significantly underestimate the measured value,'star' indicates *P*<0.01 when compared with measured creatinine clearance (CL_CR_). The modified Cockcroft-Gault (modCG) formula performs the most poorly in this setting. CG, Cockcroft-Gault; MDRD_4, 4-variable modification of diet in renal disease equation; MDRD_6, 6-variable modification of diet in renal disease equation.

A direct comparison between each assessment technique is presented in Table 3 and graphically in Figure 1. As demonstrated, each mathematical estimate was significantly lower than the median measured CL_CR _value. Although a statistically significant correlation was noted between CL_CR _and CG (*P *= 0.017), modified CG (*P *= 0.044) and 4-variable MDRD (*P *= 0.047) estimates, the strength of these correlations was poor, with Spearman coefficients (r_s_) less than 0.3. The modified CG estimates demonstrate better correlation in the Portugal cohort (*P *= 0.017), although this remains very weak (r_s _= 0.36). Using a cut-off for ARC of more than 130 ml/min/1.73 m^2^, CG estimates had the greatest sensitivity, correctly identifying 53 (62%) of the cohort. The 4-variable and 6-variable MDRD formulae were less accurate, with sensitivities of 47% and 29%, respectively (see Figure 1 andTable 3).

**Table 3:** Correlation between different measures of glomerular filtration

	Median (IQR) (All, *n *= 86)	r_s _(*P*-value) (All, *n *= 86)	r_s _(*P*-value) (Portugal, *n *= 43)	r_s _(*P*-value) (Australia, *n *= 43)
Measured CL_CR_, ml/min/1.73 m^2^	162 (145-190)			
CG, ml/min/1.73 m^2^	135 (116-171)*	0.26 (0.017)	0.29 (0.059)	0.29 (0.056)
Modified CG, ml/min/1.73 m^2^	93 (83-110)*	0.22 (0.044)	0.36 (0.017)	0.05 (0.732)
4-variable MDRD, ml/min/1.73 m^2^	124 (102-154)*	0.22 (0.047)	0.22 (0.161)	0.24 (0.122)
6-variable MDRD, ml/min/1.73 m^2^	108 (87-135)*	0.18 (0.097)	0.25 (0.105)	0.11 (0.490)

* *P*<0.01 when compared with measured CL_CR_.

CG, Cockcroft Gault; CL_CR_, creatinine clearance; IQR, interquartile range; MDRD, modification of diet in renal disease; r_s _= Spearman correlation coefficient.

**Figure 2:**
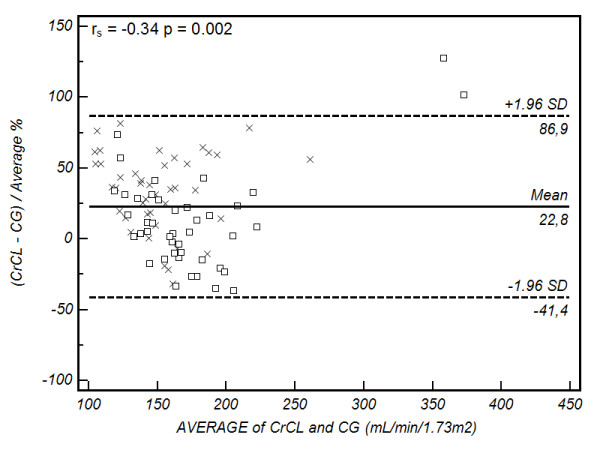
**Bland-Altman plot of CL_CR _vs Cockcroft Gault formula**. Comparison of the difference between the measured creatinine clearance (CL_CR_) and Cockcroft Gault (CG) formula (as a percentage) on the y-axis, versus the average value obtained on the x-axis. The solid line represents the bias (mean percentage difference obtained across the range of values), where as the dashed lines are the limits of agreement (+/- 1.96 × standard deviation (SD)). square, Australia cohort; cross, Portugal cohort. The Spearman correlation coefficient (r_s_) for the percentage difference and average value is provided in the top left hand corner (outliers excluded).

**Figure 3:**
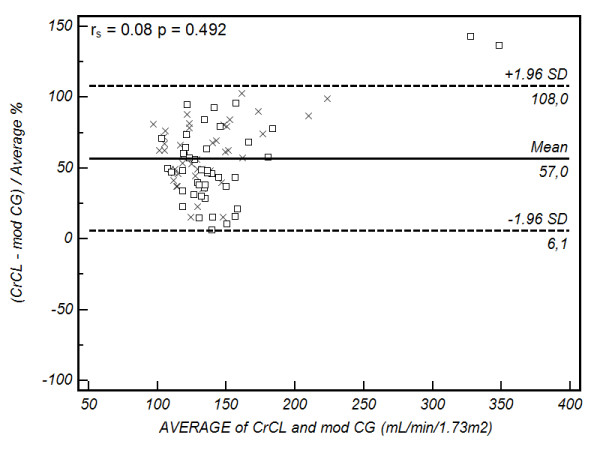
**Bland-Altman plot of CL_CR _vs modified Cockcroft Gault formula**. Comparison of the difference between the measured creatinine clearance (CL_CR_) and modified Cockcroft Gault (modCG) formula (as a percentage) on the y-axis, versus the average value obtained on the x-axis. The solid line represents the bias (mean percentage difference obtained across the range of values), where as the dashed lines are the limits of agreement (+/- 1.96 × standard deviation (SD)). square, Australia cohort; cross, Portugal cohort. The Spearman correlation coefficient (r_s_) for the percentage difference and average value is provided in the top left hand corner (outliers excluded).

**Figure 4:**
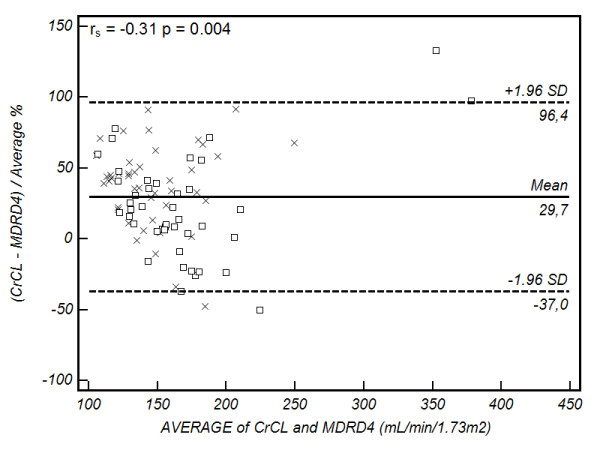
**land-Altman plot of CL_CR _vs 4-variable modification of diet in renal disease equation**. Comparison of the difference between the measured creatinine clearance (CL_CR_) and 4-variable modification of diet in renal disease equation (MDRD_4) (as a percentage) on the y-axis, versus the average value obtained on the x-axis. The solid line represents the bias (mean percentage difference obtained across the range of values), where as the dashed lines are the limits of agreement (+/- 1.96 × standard deviation (SD)). square, Australia cohort; cross, Portugal cohort. The Spearman correlation coefficient (r_s_) for the percentage difference and average value is provided in the top left hand corner (outliers excluded).

**Figure 5:**
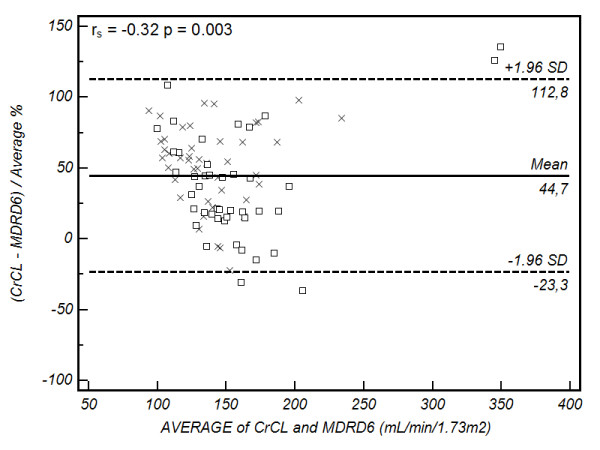
**Bland-Altman plot of CL_CR _vs 6-variable modification of diet in renal disease equation**. Comparison of the difference between the measured creatinine clearance (CL_CR_) and 6-variable modification of diet in renal disease equation (MDRD_6) (as a percentage) on the y-axis, versus the average value obtained on the x-axis. The solid line represents the bias (mean percentage difference obtained across the range of values), where as the dashed lines are the limits of agreement (+/- 1.96 × standard deviation (SD)). square, Australia cohort; cross, Portugal cohort. The Spearman correlation coefficient (r_s_) for the percentage difference and average value is provided in the top left hand corner (outliers excluded).

Bland-Altman plots are presented in Figures 2, 3, 4 and 5. Summary values for each equation overall and at each centre separately are presented in Table 4. As demonstrated, all of the formulae had poor clinical utility in terms of their precision and bias, although CG estimates appeared to perform better in the Australian setting. Examining the relation between the observed difference (as a percentage) and the average value, weak correlations were identified for CG (r_s _= -0.34, *P *= 0.002), 4-variable MDRD (r_s _= -0.31, *P *= 0.004), and 6-variable MDRD (r_s _= -0.32, *P *= 0.003) estimates, suggesting a small negative proportional error. No correlation was identified with the modified CG formula (see Figures 2, 3, 4 and 5 and Table 4).

**Table 4:** Precision and bias between measured CL_CR _and mathematical estimates

	All patients(*n *= 86)		Portugal patients (*n *= 43)		Australia patients (*n *= 43)	
	**Bias**	**Precision**	**Bias**	**Precision**	**Bias**	**Precision**

CG, ml/min/1.73 m^2^, (%)	39 (23)	± 75 (33)	50 (34)	± 47 (28)	28 (12)	± 96 (34)
Modified CG, ml/min/1.73 m^2^, (%)	84 (57)	± 70 (26)	83 (61)	± 42 (21)	85 (53)	± 93 (30)
4-variable MDRD, ml/min/1.73 m^2^, (%)	48 (30)	± 76 (34)	56 (38)	± 52 (30)	41 (22)	± 97 (36)
6-variable MDRD, ml/min/1.73 m^2^, (%)	68 (45)	± 76 (35)	73 (52)	± 48 (29)	63 (37)	± 99 (38)

CG, Cockcroft Gault; CL_CR_, creatinine clearance; MDRD, modification of diet in renal disease.

There was no significant correlation between fluid balance (r_s _= 0.16, *P *= 0.13) or acute physiology and chronic health evaluation (APACHE) II score (r_s _= 0.03, *P *= 0.776) and the measured CL_CR_. Although the daily fluid balance was considerably more negative in those who received diuretics (-541 (1207) ml vs 895 (1651) ml, *P*<0.001), there was no significant difference in CL_CR _(158 (141-179) vs 164 (147-208) ml/min/1.73 m^2^, *P *= 0.20). Neither fluid balance (*P *= 0.31), nor CL_CR _(*P *= 0.17) were significantly different between diagnostic categories, and there was no difference in CL_CR _in those receiving vasoactive medications (159 (141-169) vs 166 (150-196) ml/min/1.73 m^2^, *P *= 0.11).

## Discussion

Our results demonstrate that in critically ill patients exhibiting ARC, mathematical estimates of GFR are insensitive in identifying this phenomenon. Clinicians will often consider renal function in both their choice and dose of pharmaceuticals, in particular antibacterial agents. For example, using a previously published population pharmacokinetic model of vancomycin in the critically ill [6], required daily dosing could vary by as much as 1000 mg when using estimated versus measured values. Significantly, lower dose selection could predispose to sub-therapeutic drug exposure, treatment failure or the selection of drug-resistant strains [7], and as such, clinicians should be cautious when employing such estimates of GFR in this setting.

Ours is not the first study to raise concerns about the validity of these equations in the non-CKD population. Herrera-Gutierrez *et al. *in their work comparing 2-hour versus 24-hour CL_CR _measurements in the ICU, also examined the accuracy of Cockcroft-Gault estimates [8]. In 359 recently admitted patients, the mean 24-hour CL_CR _was 100.9 ± 4.21 ml/min/1.73 m^2^, as compared with 87.4 ± 3.05 ml/min/1.73 m^2 ^when determined by Cockcroft-Gault [8]. The reported bias was 21.87 ml/min/1.73 m^2 ^with a precision of ± 58.27 ml/min/1.73 m^2^. Importantly, this was largely generated by those patients with a CL_CR _of more than 100 ml/min/1.73 m^2 ^[7], and compares favourably with our study. A similar result was also noted by Martin *et al. *in 109 critically ill patients, where only a weak correlation was demonstrated between 24-hour measured CL_CR _and Cockcroft-Gault estimates [9].

Cherry *et al. *have also examined measured CL_CR _versus mathematical estimates in a cohort of critically ill and traumatised patients. In 100 patients (45 trauma victims), Cockcroft-Gault estimates significantly underestimated the mean 24-hour CL_CR _(CL_CR _= 103.2 ± 5.7 ml/min vs CG CL_CR _= 86.2 ml/min ± 4.2) [10], although separate investigators have suggested a modified Cockcroft-Gault equation is reliable in stable trauma patients [11]. In comparison, although approximately 60% of the patients in this study were victims of trauma, significant numbers required mechanical ventilation, vasoactive medications or were septic on the day of the study.

Hoste *et al. *examined the relation between a measured 1-hour CL_CR _and Cockcroft-Gault, 6-variable and 4-variable MDRD estimates in recently admitted critically ill patients with normal serum creatinine concentrations [12]. Twenty-eight older (median age 58 years) moderately sick (median APACHE II 21) patients were included, with a measured CL_CR _of 86 (62.6-121.6) ml/min/1.73 m^2 ^[12]. Of note, only the 6-variable MDRD equation had any degree of statistical correlation with the measured value (R = 0.466, *P *= 0.012), and biases were much lower than reported in our study (Cockcroft-Gault -6.2, 6-variable MDRD 11.2, 4-variable -9.4 ml/min/1.73 m^2^) [12]. Importantly, a significant number of these patients (*n *= 13) had renal impairment (CL_CR _<80 ml/min/1.73 m^2^), despite a normal serum creatinine concentration. This is in agreement with data provided by Poggio *et al. *noting similar levels of bias in ill hospitalised patients with moderate renal dysfunction, as compared with iothalamate measures of GFR [13].

More recently, Martin *et al. *have examined the use of MDRD and Cockcroft-Gault estimates in a cohort of primarily head injured or burnt patients with normal serum creatinine concentrations. Measured 8-hour CL_CR _values were significantly elevated (median 163 (124-199) ml/min), and substantial bias was reported with both mathematical formulae (-12 ml/min/1.73 m^2 ^4-variable MDRD, 17 ml/min Cockcroft-Gault CL_CR_) [14]. Of note, significant improvement in MDRD performance was seen with correction for anthropomorphic measures [14]. Conil *et al. *have also noted the pitfalls of using such equations in patients with burn injuries, reporting a mean measured 24-hour CL_CR _of 119 ± 53 ml/min/1.73 m^2^, compared with 98 ± 38 ml/min/1.73 m^2^, and 101 ± 52 ml/min/1.73 m^2 ^with 4-variable MDRD and Cockcroft-Gault estimates, respectively [15]. A significant negative bias was noted with both equations.

These data confirm that these commonly employed estimates of GFR are largely flawed in the critically ill, and should be viewed with caution in this setting. Our study extends this prior work, with analysis in a selected population of patients exhibiting ARC (CL_CR _>130 ml/min/1.73 m^2^). Although a relatively new term, ARC reflects supra-normal renal excretion of circulating solute [16], and is being increasingly recognised in the ICU environment [17,18], largely as a consequence of the underlying inflammatory state and therapeutic interventions provided [19]. Of note, the sub-group manifesting ARC in our analysis were primarily young male traumatised patients, and is in keeping with recent work by Minville *et al*., demonstrating elevated CL_CR _in polytrauma victims [20].

The implications of this phenomenon primarily relate to the potential for sub-therapeutic drug exposure, and treatment failure. This is reinforced by research demonstrating a close correlation between drug elimination and CL_CR _[21,22], in addition to data provided by the Chronic Kidney Disease Epidemiology Collaboration (CKD-EPI), demonstrating that mathematical estimates of GFR can result in up to about 20% discordance in drug-dosing recommendations, depending on the equation employed [23]. This is likely to be even higher in those manifesting ARC, because the population reported had significantly lower measured GFRs (mean (standard deviation) GFR-75 (44) ml/min) [23], compared with those observed in this analysis.

This study has a number of potential limitations. Firstly, it represents a *post-hoc *analysis of prospectively collected data. Secondly, an 8-hour CL_CR _was employed in Australia, while a 24-hour collection was performed in Portugal, although previous authors have demonstrated acceptable agreement when using either technique [10,24]. Importantly, our data demonstrate that mathematical estimates have poor clinical utility in comparison to either measure. Thirdly, calibration of creatinine assays can also introduce systematic bias, but as both laboratories use the same analytical process, this should be limited. Fourthly, it could be considered that our patients were not at 'steady-state' and as such, the serum creatinine concentrations are systematically lower than might be expected. However, there was no significant correlation between fluid balance and CL_CR_, and vasoactive medications, diuretic administration, and admission diagnosis had no influence on the measured value. Finally, although CL_CR _is not considered a gold standard measure of GFR (due to tubular secretion of creatinine at lower filtration rates) [25], in the population under study (CL_CR _>130 ml/min/1.73 m^2^), this is unlikely to be a major cause of error.

Examining our data closely, two patients appeared to have CL_CR _values that were well outside the 'normal' range, and as such, lack biological plausibility (Figures 2, 3, 4 and 5). These 'outliers' likely represent a random error in measurement, although on repeated inspection, no specific fault could be identified. These results are reported in order to maintain the integrity of the dataset, but must be viewed with caution. Repeating the analysis after removing these values (*n *= 84), continued to demonstrate clinically unacceptable bias and precision (Cockcroft-Gault CL_CR _30 ± 47 ml/min/1.73 m^2^, modified Cockcroft-Gault CL_CR _75 ± 39 ml/min/1.73 m^2^, 4-variable MDRD 40 ± 52 ml/min/1.73 m^2^, and 6-variable MDRD 59 ± 49 ml/min/1.73 m^2^) as compared with the measured values.

## Conclusions

In conclusion, this study has demonstrated that commonly employed estimates are inaccurate in quantifying GFR in a sub-group of critically ill patients with ARC. Both Cockcroft-Gault and MDRD derived values significantly underestimate the measured CL_CR _and are insensitive in identifying this phenomenon. This has important ramifications for adequate dosing of various pharmaceuticals in this setting, particularly antibacterial agents. Clinicians should be cautious in altering prescriptions on the basis of mathematical estimates alone. Instead we recommend the routine use of measured CL_CR _as a surrogate of GFR in the ICU.

## Key messages

• A significant proportion of critically ill patients will have creatinine clearances well above the normal reference range, a phenomenon termed ARC.

• Creatinine clearance is closely correlated with renal drug elimination.

• Mathematical estimates of GFR and creatinine clearance are flawed in the critically ill, and will tend to significantly under-estimate renal function in those with ARC.

• Altering drug prescription on the basis of these estimates is likely to lead to sub-therapeutic drug concentrations, promoting the possibility of treatment failure.

## Abbreviations

APACHE: acute physiology and chronic health evaluation; ARC: augmented renal clearance; CKD: chronic kidney disease; CL_CR_: creatinine clearance; eGFR: estimated glomerular filtration rate; GFR: glomerular filtration rate; MDRD: modification of diet in renal disease equation; SIRS: systemic inflammatory response syndrome.

## Competing interests

Dr Baptista has previously acted as a consultant for AstraZeneca. Dr Udy has no conflicts of interest to disclose. Dr Sousa has no conflicts of interest to disclose. Professor Pimentel has no conflicts of interest to disclose. Miss Wang has no conflicts of interest to disclose. Dr Roberts has no conflicts of interest to disclose.

Professor Lipman is a consultant to AstraZeneca and Janssen-Cilag, and has received an honorarium from AstraZeneca, Janssen-Cilag and Wyeth Australia. AstraZeneca provides an annual donation to the Burns, Trauma and Critical Care Research Center (BTCCRC), University of Queensland.

## Authors' contributions

JB, AU, LW, ES, JR and JP were involved in protocol development, ethics approval and implementation. JB, AU and LW were involved in data acquisition. JB and AU performed the statistical analysis. JB, AU, JR and JL wrote the manuscript. AU takes responsibility for archiving the data and guarantees the integrity of the paper form inception to publication. All of the authors have read and approved the article for publication.
